# Beyond the *emm*-type: fine-tuning Group A *Streptococcus* typing with Enn and Mrp

**DOI:** 10.1128/spectrum.02047-25

**Published:** 2025-11-14

**Authors:** C. Widomski, C. Bruyns, L. Schiavolin, G. de Crombrugghe, P. R. Smeesters, A. Botteaux

**Affiliations:** 1 Molecular Bacteriology, Laboratory of Microbiology, European Plotkin Institute for Vaccinology (EPIV), Université libre de Bruxelles 129212, Brussels, Belgium; 2 Department of Paediatrics, Brussels University Hospital, Academic Children Hospital Queen Fabiola, Université libre de Bruxelles 129212, Brussels, Belgium; The Ohio State University College of Dentistry, Columbus, Ohio, USA

**Keywords:** PCR, M-like proteins, M protein, *emm*-typing, vaccine, Group A *Streptococcus*

## Abstract

Regarding the increased tendency to look at the M and M-like proteins as a functional trio, we set up a PCR-based typing of the *enn* and *mrp* genes in Group A *Streptococcus* (GAS). Based on a large *mga* regulon database, we have designed specific PCR primers for amplification and sequencing of *enn* and *mrp* genes. Primers were first validated *in silico* using a global database of 1,419 GAS genomes and then *in vitro* on 17 isolates covering all subclusters of Enn and Mrp proteins. Off-targets were also checked against other streptococci harboring M-like genes. We also described how to assign new Enn- and Mrp-types based on an up-to-date database available online. This new typing system will enhance the precision of strain characterization and help understand some differences between isolates sharing the same M-type.

**IMPORTANCE:**

*Streptococcus pyogenes*, also known as Group A *Streptococcus*, is a bacterium that causes a wide range of diseases, from mild sore throats to life-threatening infections and long-term complications like rheumatic heart disease. It is responsible for over 500,000 deaths a year. For decades, scientists have focused on the surface M protein which helps it survive in human tissues. The large diversity of its N-terminal region has led to the *emm*-typing system of *S. pyogenes*. However, the two M-like proteins, Enn and Mrp, produced by the same region of the genome, have been largely overlooked until recently. These proteins are found in many strains around the world, and recent findings suggest they also play an important role in *S. pyogenes* virulence. In our study, we developed a new typing system for Enn and Mrp proteins that aims to better understand their behavior and improve the monitoring of clinical strains.

## INTRODUCTION

*Streptococcus pyogenes* or Group A *Streptococcus* (GAS) is a leading global pathogen responsible for more than 500,000 deaths a year (1). Its virulence is due to an arsenal of virulence factors that varies between strains (2). Strains are categorized into *emm*-types by the sequence of the hypervariable 5′ region (HVR) of the *emm* gene (3). The *emm* gene encodes a surface-exposed protein, the M protein, which is a major virulence factor and the target of protective immune response (2). The *emm* type is believed to be predictive of the genomic content. However, differences in virulence are observed among strains sharing the same *emm*-type (4). To date, over 270 different *emm*-types have been identified.

**Fig 1:**
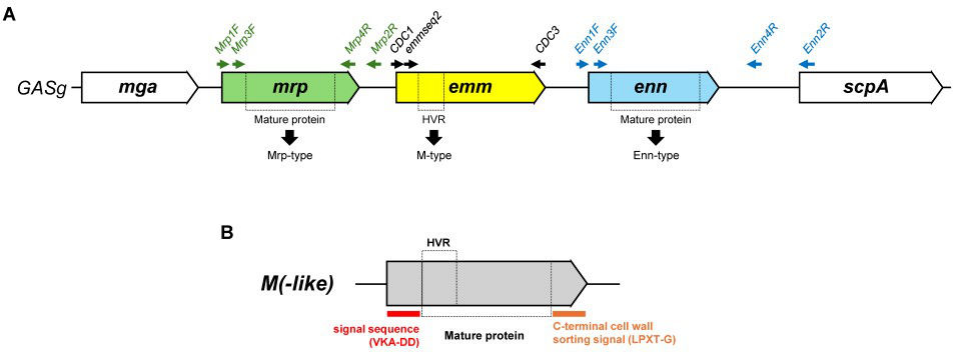
Schematic representation of the Mga core regulon containing *mga*, *mrp*, *emm*, *enn*, and *scpA* genes. Amplification and sequencing primers for *emm* are depicted as black arrows, for *mrp* as green arrows and for *enn* as blue arrows (**A**). The difference between *emm*-type based on the nucleotide sequence of the HVR region and the Enn- and Mrp-types, which are based on the amino acid sequence of the mature protein, is schematically explained in (**B**). The signal sequence (red) and cell wall sorting signal (orange) are cleaved (hyphen between amino acids) to generate the exposed mature protein.

The worldwide database of *emm*-type specific sequences is hosted and curated by the U.S. Centers for Disease Control and Prevention (https://www.cdc.gov/strep-lab/php/group-a-strep/emm-typing.html). Sequences are generated either by PCR amplification and sequencing using primers located in conserved regions of the signal peptide and the 5′ end of the *emm* gene (Fig. 1), or by *de novo* assembly of whole-genome sequencing (WGS) data. Recently, our team has proposed a solution to solve the most common problem with *emm*-typing, that is, double bands due to similarity in sequence between the *emm* genes and two closely related *emm*-like genes called *mrp* and *enn*, by designing a new amplifying primer, called CDC3 (3).

WGS studies allow describing the *mga* regulon, which contains *emm* but also the *emm*-like genes *mrp* and *enn* (5). These genes are present in more than 85% of the circulating strains, and mainly in strains from low- and middle-income settings. These M-like proteins share structural and binding capacities with the M protein, but their exact role in virulence is not fully understood (5). Genetic diversity of the *enn* and *mrp* was recently investigated and showed 352 *enn* and 295 *mrp* unique alleles (6). These alleles encode 276 Enn and 225 Mrp mature proteins (after the signal peptide up to the threonine residue of the LPXTG-sortase motif) (6). By contrast to *emm*-typing, numbering of Enn and Mrp variants was done on the amino acid sequence of the entire mature protein (Fig. 1). These proteins were then divided into 9 Enn and 10 Mrp phylogenetic sub-groups (SG) (6).

We propose a novel Enn- and Mrp-typing protocol, complementary to the *emm*-typing, which will inform on the Mga core regulon content and would be more predictive of the strain behavior.

## MATERIALS AND METHODS

Primer-3 package in Geneious Prime software (V2025.01) was used to predict primer binding sites and properties using default *in silico* PCR parameters. Off-target primer annealing was verified using Geneious on available *Streptococcus* spp. genomes harboring emm-like genes (*Streptococcus dysgalactiae* subsp. *equisimilis*, *Streptococcus dysgalactiae* subsp. *dysgalactiae*, and *Streptococcus equi* subsp. *zooepidemicus*) from NCBI. PCR was performed in 50 µL total volume using GoTaq (Promega) polymerase and buffer, containing 1.5 mM MgCl_2_, 0.2 mM each dNTP, 10 nM forward and reverse primers, and sterile distilled water. GAS DNA was prepared with InstaGene Matrix (Bio-Rad) according to the manufacturer’s instructions. Primers used are listed in Table S1. The following thermal cycling conditions were applied: denaturation at 94°C for 1 min, 30 cycles of 94°C for 15 s, annealing at 42°C (*enn*) or 55°C (*mrp*) for 30 s, extension at 72°C for 1 min 25 s, and final elongation at 72°C for 5 min. PCR products were visualized on 1% agarose gels stained with SafeRed dye (Carl Roth) with migration at 200 V for 20 min.

The PCR products of 17 isolates were purified using the Monarch PCR & DNA Cleanup Kit (NEB) and sequenced using cycle sequencing on an Applied Biosystems 3730XL instrument by Eurofins Genomics (https://www.eurofinsgenomics.eu/en/home/) with the appropriate sequencing primers (Tables S1 and S2). Sequences were analyzed with Geneious Prime software (V2025.01) by blast against a database of 307 Enn and 254 Mrp.

## RESULTS

Alignment of 1,419 *mga* regulons (7) allowed us to design specific primers for *enn* (Enn1F and Enn2R) and *mrp* (Mrp1F and Mrp2R) amplification (Table S1). Forward primer for *enn* PCR was designed in the intergenic region between *emm* and *enn*, while reverse primer was designed in the 5′ end of the *scpA* gene (Fig. 1). Forward primer for *mrp* PCR was designed in the intergenic region between *mga* and *mrp*, while reverse primer was designed in the 5′ end of the *emm* gene (Fig. 1).

Primer annealing was checked *in silico* using the Primer-3 package in Geneious Prime. Amplification primers were specific and annealed on all the available genomes containing *enn* and *mrp* genes in our database. However, Enn1F/Enn2R and Mrp1F/Mrp2R primer pairs did not allow for the amplification of some *enn* or *mrp* genes associated with a limited number of M-types (Table S3) (8).

We then tested both primer pairs *in vitro* in 16 strains (Table S4) containing *enn* and *mrp* genes from each known cluster (6, 9–12). An M5 strain was used as a negative control since it has no *enn* and *mrp* gene.

**Fig 2:**
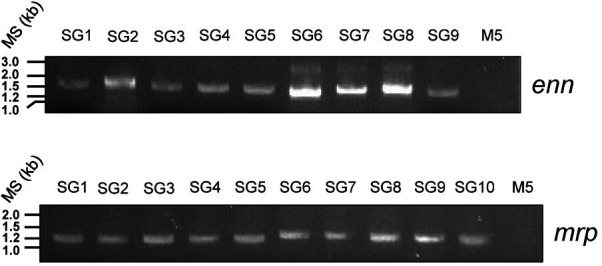
Amplification of *enn* and *mrp* genes in GAS. *enn* and *mrp* genes were amplified by PCR using Enn1F/Enn2R and Mrp1F/Mrp2R, respectively. Amplicon of *enn* (upper panel) or *mrp* (lower panel) was separated on a 1% agarose gel and visualized under UV. kb, kilobases; MS, molecular size.

After genomic DNA preparation, PCR was performed using Enn1F/Enn2R and Mrp1F/Mrp2R primers (Fig. 2). The expected size of the PCR products ranges from 1,291 to 1,681 bp for *enn* and from 1,153 to 1,613 bp for *mrp*. For most PCR reactions, we observed a single band of the expected size. However, in approximately three out of nine Enn SG samples, an additional band of higher molecular size was detected. Adjusting the annealing temperature did not eliminate this non-specific band. The band at around 1.2 kb was extracted from the agarose gel, sequenced, and identified as the *enn* gene. The band of higher molecular weight size is a non-specific amplification.

PCR products were then sequenced by Eurofins using Enn3F-4R for *enn* genes. Sequencing of *mrp* genes was done using Mrp3F as the forward primer and MrpB4R or MrpA5R as the reverse primer depending on the clade (A or B) they belong to. Table S2 lists the *emm*-type generally associated with each Mrp clade in order to select the appropriate primer. These primer pairs allow the sequencing of a portion of the gene encoding for the mature proteins. After translation, the sequences were analyzed using BLAST against our Enn and Mrp proteins database to assign a number and an SG (available at https://zenodo.org). Any change in one amino acid in the mature protein sequence defines a new Enn or Mrp-type (Fig. 1). Sequences of new Enn or Mrp type should be submitted to GAS-typing.EPIV@ulb.be for number and SG attribution.

## DISCUSSION

Our data support the establishment of the first Enn-typing and Mrp-typing protocol for molecular characterization of *S. pyogenes*. We have previously shown that the M/Enn/Mrp association is not random (6), which provides preliminary evidence of the existence of a functional basis for their co-inheritance. Growing evidence shows that M, Mrp, and Enn share common features and act in partnership to escape from the immune system (13, 14). The combination of *emm*-typing using CDC1/CDC3 (3), Enn- and Mrp-typing will enhance our understanding of their specific association and help in understanding their role as a trio of surface proteins in GAS virulence.

The sequences of the proposed primers are based on the most conserved and specific regions. These primers are theoretically capable of amplifying more than 97.8% and 99.5% of known *enn* and *mrp* alleles, respectively. The use of this new protocol will determine the real proportion of GAS strains that will potentially remain non-typeable. In cases of untypeable *emm* genes and/or based on known associations between M-types and *mrp/enn* alleles (6), the presence of a particular *mrp* or *enn* allele can be inferred and should be further investigated when PCR amplification fails. These should then be investigated using next-generation sequencing for confirmation.

Assignment of the Mrp/Enn-type can be performed by aligning the mature protein extracted from the sequenced PCR product with the “Typing” database available on https://zenodo.org (https://doi.org/10.5281/zenodo.17270970 and https://doi.org/10.5281/zenodo.17491344). In the case of new Mrp/Enn-type, the authors are encouraged to send their raw sequencing data to the GAS-typing.EPIV@ulb.be email address. This holds true for new M/Enn/Mrp associations not found in the “association” database available on https://zenodo.org (https://doi.org/10.5281/zenodo.17491475). Both databases will be updated on a regular basis.

## SUPPLEMENTAL MATERIAL

Supplemental material spectrum.02047-25-s0001.docx Tables S1 to S4.

ASM does not own the copyrights to Supplemental Material that may be linked to, or accessed through, an article. The authors have granted ASM a non-exclusive, world-wide license to publish the Supplemental Material files. Please contact the corresponding author directly for reuse.
