# Supplementary material for: Beyond the emm-type: fine-tuning Group A Streptococcus typing with Enn and Mrp
Source: Microbiol Spectr. 2025 Nov 14;14(1):e02047-25. doi: 10.1128/spectrum.02047-25 (PMC12772289; doi:10.1128/spectrum.02047-25)
Supplement: Supplemental material — Tables S1 to S4. [file spectrum.02047-25-s0001.docx]

Table S1. Primers used in this study.

| **Primer name** | **Primer sequence** |
| --- | --- |
| Enn1F | 5’-ATAAAATYAAGGAGYAGATAA-3’ |
| Enn2R | 5’-CATTGAGCAAGATGCTCGTAGACA-3’ |
| Enn3F | 5’-CTAGACAACAAACCAAGAAAAATT-3’ |
| Enn4R | 5’-GTTATTTGCATATGATACAGCT-3’ |
| Mrp1F | 5’-CTACTCAYTGAGAAAGTTAAMAACAGGTAC-3’ |
| Mrp2R | 5’-GTAAAGAYCAGGTTTTTGAGACTTTAGT-3’ |
| Mrp3F | 5’-TCAGTAGCAGTAGCTTTG-3’ |
| MrpB4R | 5’-GCTGCAGTGAAGAATGGG-3’ |
| MrpA5R | 5’-TTGCGTTTTAGGGCAAGT-3’ |

Table S2. List of *emm*-types associated with Mrp from clade A or B. SG: sub-groups. Emm-type in bold can be associated to either a *mrp* from clade A or clade B.

| Clade A (SG1-2-3-4-5-10) | Clade B (SG6-7-8-9) |
| --- | --- |
| M4-**8**-**15**-18-**22**-25-**28**-32-33-36-41-**42**-43-48-52-53-54-56-58-60-**63**-64-**65**-68-71-74-**75**-**78**-80-81-82-83-84-86-90-93-95-97-98-100-101-103-**104-**105-108-109-111-**113**-115-**116**-117-**118**-119-122-123-165-176-178-179-184-186-192-207-209-217-218-221-223-224-225-230 | M2-**8**-9-11-**15**-**22**-23-**28**-**42**-44-49-59-**63**-**65**-66-73-**75**-77-**78**-79-85-87-88-89-94-99-102-**104**-106-110-112-**113**-114-**116**-**118**-124-177-183-185-191-232 |

Table S3. *enn* and *mrp* genes not amplified by primers used in this study.

| **Gene name** | **Associated M-type** | **% of isolates (Smeesters et al. 2024)** | **Non annealing primer** |
| --- | --- | --- | --- |
| *enn351* | - | - | Enn1F/2R |
| *enn289* | M178 | < 0.01 | Enn1F/2R |
| *enn262* | M71 | 0.21 | Enn1F/2R |
| *enn343* | M11 | 1.9 | Enn1F/2R |
| *mrp203* | - | - | Mrp1F/Mrp2R |
| *mrp209-201* | M86 | 0.13 | Mrp1F/Mrp2R |
| *mrp206* | M224 | < 0.01 | Mrp1F/Mrp2R |
| *mrp270* | M122 | 0.07 | Mrp1F/Mrp2R |
| *mrp265-266-267* | M105 | 0.13 | Mrp1F/Mrp2R |
| *mrp245* | M207 | 0.07 | Mrp1F/Mrp2R |

Table S4. Strains used in this study.

| **emm-type** | **M cluster** | **Mrp-type** | **Mrp cluster** | **Enn-type** | **Enn cluster** | **ref** |
| --- | --- | --- | --- | --- | --- | --- |
| M5 (CTRL-) | Single Y | NA | NA | NA | NA | (13) |
| M2 | E4 | 5 | SG6 | 127 | SG1 | (14) |
| M28 | E4 | 134 | SG1 | ND | ND | (15) |
| M33 | D4 | 219 | SG2 | 251 | SG5 | (14) |
| M36 | D1 | ND | ND | 255 | SG7 | (15) |
| M48 | E6 | ND | ND | 340 | SG8 | (15) |
| M58 | E3 | 126 | SG5 | 52 | SG3 | (14) |
| M68 | E2 | ND | ND | 22 | SG4 | (16) |
| M81 | E6 | ND | ND | 203 | SG6 | (16) |
| M87 | E3 | 34 | SG7 | ND | ND | (14) |
| M94 | E6 | 96 | SG8 | ND | ND | (14) |
| M97 | D5 | 284 | SG3 | ND | ND | (16) |
| M101 | D4 | ND | ND | 300 | SG9 | (15) |
| M103 | E3 | 293 | SG10 | ND | ND | (16) |
| M180 | E3 | ND | ND | 385 | SG2 | (16) |
| M183 | E3 | 67 | SG9 | ND | ND | (16) |
| M208 | D4 | 190 | SG4 | ND | ND | (16) |
